# A New Ligustrazine Derivative-Selective Cytotoxicity by Suppression of NF-κB/p65 and COX-2 Expression on Human Hepatoma Cells. Part 3

**DOI:** 10.3390/ijms160716401

**Published:** 2015-07-17

**Authors:** Chenze Zhang, Wenqiang Yan, Bi Li, Bing Xu, Yan Gong, Fuhao Chu, Yuzhong Zhang, Qiuli Yao, Penglong Wang, Haimin Lei

**Affiliations:** 1 School of Chinese Pharmacy, Beijing University of Chinese Medicine, Beijing 100102, China; E-Mails: zcz920418@163.com (C.Z.); ywq3226925@163.com (W.Y.); libimegan@163.com (B.L.); weichenxubing@126.com (B.X.); gongyan90@163.com (Y.G.); chufhao@163.com (F.C.); 2 Department of Pathology, Beijing University of Chinese Medicine, Beijing 100102, China; E-Mail: zyz100102@126.com; 3 School of Nursing, Beijing University of Chinese Medicine, Beijing 100102, China; E-Mail: yaoqiuli2008@163.com

**Keywords:** ligustrazine derivative, selective cytotoxicity, hepatoma, NF-κB/p65 and COX-2

## Abstract

A new anticancer ligustrazine derivative, 3β-hydroxyolea-12-en-28-oic acid-3,5,6-trimethylpyrazin-2-methylester (T-OA, C_38_H_58_O_3_N_2_), was previously reported. It was synthesized via conjugating hepatoprotective and anticancer ingredients of traditional Chinese medicine. We found that T-OA exerted its anticancer activity by preventing the expression of nuclear transcription factor NF-κB/p65 and COX-2 in S180 mice. However, the selective cytotoxicity of T-OA on various kinds of cell lines has not been studied sufficiently. In the present study, compared with Cisplatin, T-OA was more toxic to human hepatoma cell line Bel-7402 (IC_50_ = 6.36 ± 1.56 µM) than other three cancer cell lines (HeLa, HT-29, BGC-823), and no toxicity was observed toward Madin–Darby canine kidney cell line MDCK (IC_50_ > 150 µM). The morphological changes of Bel-7402 cells demonstrated that T-OA had an apoptosis-inducing effect which had been substantiated using 4ʹ,6-diamidino-2-phenylindole (DAPI) staining, acridine orange (AO)/ethidium bromide (EB) staining, flow cytometry and mitochondrial membrane potential assay. Combining the immumohistochemical staining, we found T-OA could prevent the expression of NF-κB/p65 and COX-2 in Bel-7402 cells. Both of the proteins have been known to play roles in apoptosis and are mainly located in the nuclei. Moreover subcellular localization was performed to reveal that T-OA exerts in nuclei of Bel-7402 cells. The result was in accordance with the effects of down-regulating the expression of NF-κB/p65 and COX-2.

## 1. Introduction

**Figure 1:**
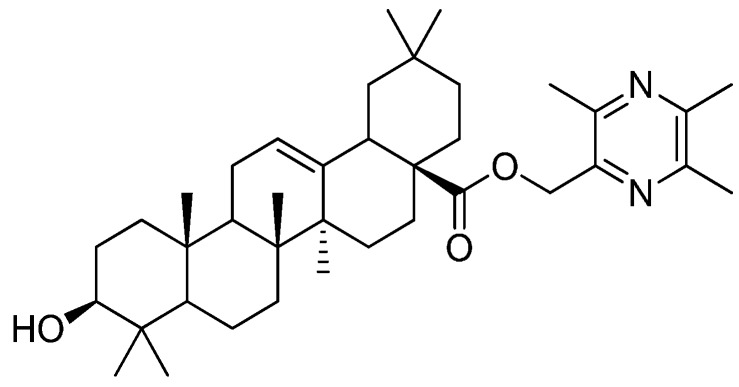
Structure of T-OA.

The attempt to apply the “combination principle” to discover lead compounds from traditional Chinese medicine (TCM) has already drawn considerable attention [1,2,3,4,5]. Ligustrazine (TMP) and oleanolic acid (OA), which are found in *ligusticum chuanxiong* and *ligustrum lucidum*, have been reported to possess anticancer activities [6,7]. In an earlier study, we synthesized T-OA (C_38_H_58_O_3_N_2_, Figure 1) by conjugating TMP and OA, and its anticancer activity was confirmed *in vitro* and *in vivo*. Further pharmacokinetic properties study also indicated that T-OA could be absorbed after single-dose oral administration. Moreover, the acute toxic test showed that LD_50_ value of T-OA exceeded 6.0 g/kg via gavage in mice [8,9,10,11,12,13].

NF-κB is a crucial transcription factor involved in regulating the balance between cell proliferation and apoptosis. It exerts effects by regulating expression chemokine, cell adhesion molecules and growth factors [14,15]. Meanwhile, NF-κB influences the expression of cyclooxygenase-2 (COX-2), which is considered a key target for anticancer therapy [16,17]. Numerous studies have demonstrated that COX-2 expression increased during the progression from normal to cancerous state [18]; COX-2 stimulates angiogenesis and is associated with tumor growth, invasion, and metastasis [19,20,21,22,23]. Moreover, when cells are stimulated with extracellular signals such as cytokines and an oxidative stressor, NF-κB translocated from the cytoplasm to the nucleus and played important roles on the anti-apoptosis effect [24]. In the previous experiments, T-OA exhibited promising anticancer effects and prevented the expression of NF-κB/p65 and COX-2 in S180 mice.

In addition, a series of previous studies proved that OA had been reported to possess hepatoprotective activity which may lead T-OA to show selectivity toward human hepatoma cells [25,26]. Moreover, both TMP and OA, the starting materials of T-OA, have earlier been documented as inhibitors of NF-κB [27,28]. Based on the above, our aim is to investigate whether T-OA exert a selective effect on human hepatoma cells and elucidate the possible mechanism of the effect.

## 2. Results and Discussion

### 2.1. In Vitro Cytotoxicity

The cytotoxicity of T-OA was evaluated by standard thiazolyl blue (MTT) assay, in comparison with Cisplatin, on Bel-7402, HeLa, HT-29, BGC-823 and MDCK cell lines. The results are the mean of the IC_50_ from the dose–response curves of three independent experiments. As shown in Table 1, the IC_50_ of T-OA for Bel-7402 cell line was nearly five times lower than that for other three cancer cell lines. Furthermore, compared with Cisplatin (positive control, IC_50_ = 3.70 ± 2.89 µM), T-OA (IC_50_ > 150 µM) showed lower renal toxicity.

**Table 1:** IC_50_ values (µM) of T-OA and Cisplatin on different cell lines.

Compound	IC_50_ Values (µM)				
	Bel-7402	HeLa	HT-29	BGC-823	MDCK
T-OA	6.36 ± 1.56	28.73 ± 5.89	29.84 ± 6.73	>30	>150
Cisplatin	5.94 ± 1.48	6.22 ± 2.06	6.11 ± 2.31	6.82 ± 1.79	3.70 ± 2.89

**Figure 2:**
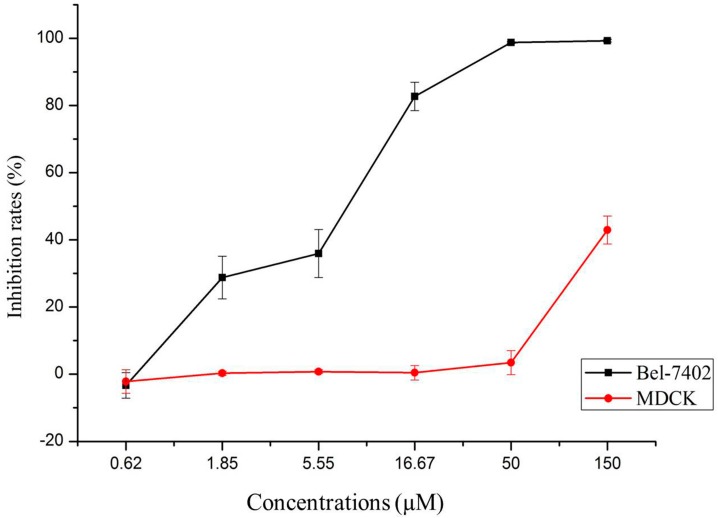
Selective cytotoxicity of T-OA on Bel-7402 and MDCK cell lines.

As shown in Figure 2, T-OA showed significant cytotoxicity toward Bel-7402 cell lines, while no toxicity was observed toward MDCK cell line at the same concentrations used with the cancer cells.

To investigate the selective effect of T-OA on human hepatoma cells and explore its possible mechanism, Giemsa staining, DAPI staining, AO/EB staining, apoptosis analysis, immunohistochemistry analysis and subcellular location were performed in the continuing research.

### 2.2. Giemsa Staining

**Figure 3:**
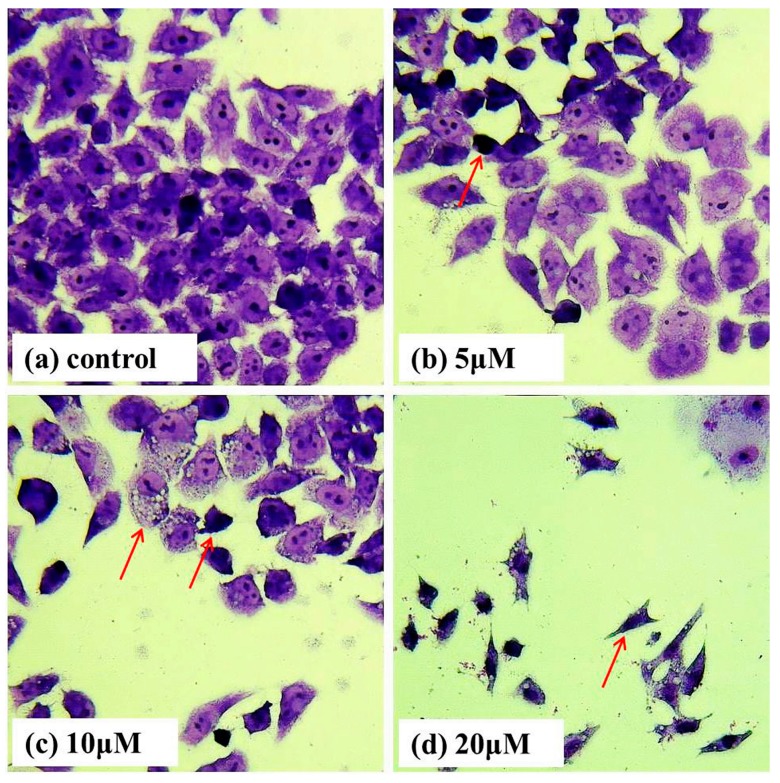
Morphological changes of Bel-7402 cells assessed by Giemsa staining. (**a**) Control group without T-OA; (**b**) Treated with 5 µM T-OA; (**c**) Treated with 10 µM T-OA; and (**d**) Treated with 20 µM T-OA. (×400).

To examine whether the loss in cell viability could be associated with the occurrence of apoptosis, we treated Bel-7402 cells with T-OA of various concentrations for 48 h and then performed Giemsa staining. Cells treated with T-OA showed obviously apoptotic characteristics (Figure 3), including cytoplasmic shrinkage, nuclear condensation, nuclear fragmentation and the formation of apoptotic bodies. Arrowheads indicate the apoptotic cells.

### 2.3. DAPI Staining

**Figure 4:**
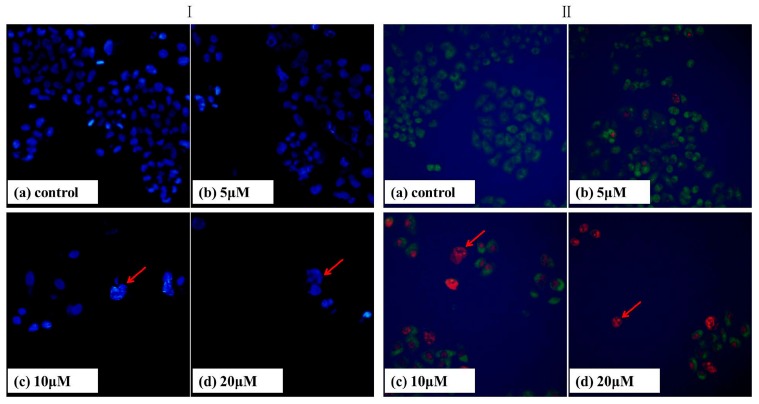
DAPI (**I**) and acridine orange/ethidium bromide (AO/EB) (**II**) staining of the Bel-7402 cells treated by T-OA. (**Ia**) Control group without T-OA; (**Ib**) Treated with 5 µM T-OA; (**Ic**) Treated with 10 µM T-OA; (**Id**) Treated with 20 µM T-OA; (**IIa**) Control group without T-OA; (**IIb**) Treated with 5 µM T-OA; (**IIc**) Treated with 10 µM T-OA; (**IId**) Treated with 20 µM T-OA. (×200)*.*

Apoptosis can be differentiated from necrosis by their characteristic nuclear changes. DAPI is a nuclear stain which is observed as blue fluorescence when excited under fluorescence microscope [29]. We treated Bel-7402 cells with T-OA of various concentrations for 48 h and then DAPI staining was performed. The control group showed intact cell bodies with clear round nuclei, while treated cells clearly showed condensed chromatin, nuclear fragmentation and weak fluorescence compared to the control cells (Figure 4I). Meanwhile, we could clearly observe that nuclear fragmentation of Bel-7402 cells increased significantly with increasing concentration of T-OA. Thus, DAPI staining indicated that T-OA could induce Bel-7402 apoptosis via nuclear fragmentation.

### 2.4. AO/EB Staining

Acridine orange (AO) and ethidium bromide (EB) are fluorescent intercalating DNA dyes. AO can stain nuclear DNA across an intact cell membrane, while EB is only taken by cells that had lost their membrane integrity. Therefore, after being stained with AO and EB, live cells will be stained green and regular-sized while late apoptotic and necrotic cells will be stained red.

As shown in Figure 4II, Bel-7402 cells were treated with T-OA of various concentrations for 48 h, and followed by AO/EB staining. Compared with the control group, the changes on the cell morphology can be obviously observed. The nuclei clearly stained as red, displayed pycnosis, membrane blebbing and cell budding (Figure 4IIc,d). This suggested cell apoptosis induction of T-OA on Bel-7402 cells, which was consistent with the previous results for Giemsa/DAPI staining.

### 2.5. Apoptosis Analysis

**Figure 5:**
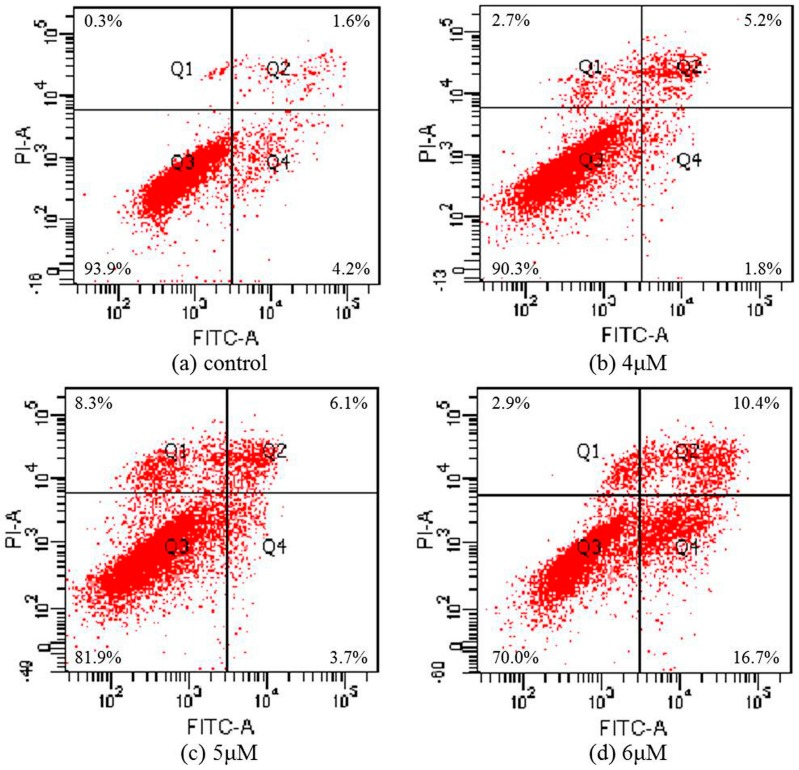
Apoptosis ratio detection by Annexin V/PI assay on the Bel-7402 cells treated by T-OA. (**a**) Control group without T-OA; (**b**) Treated with 5 µM T-OA; (**c**) Treated with 10 µM T-OA; (**d**) Treated with 20 µM T-OA.

To further confirm that T-OA can induce cell apoptosis in Bel-7402 cells, cells treated with three concentrations of T-OA were double stained with annexin V-FITC/PI, then the degree of apoptosis was measured by flow cytometry. The assay showed that T-OA treatment caused a significant increase in apoptosis. As shown in Figure 5 after 48 h of treatment, apoptosis ratios (including the early and late apoptosis ratios) were found to increase from 7% (4 µM) to 9.8% (5 µM) and 27.1% (6 µM), respectively, while that of the control was 5.8%. The results indicated that TOA could mainly induce apoptosis in Bel-7402 cells.

### 2.6. Mitochondrial Membrane Potential Assay

**Figure 6:**
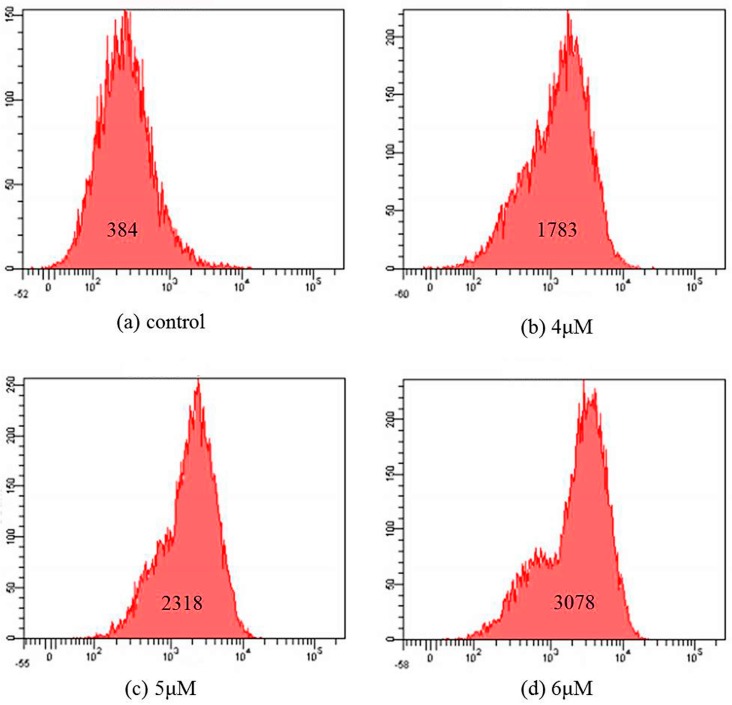
Mitochondrial membrane potential detection by Rhodamine 123 staining on the Bel-7402 cells treated by T-OA. (**a**) Control group without T-OA; (**b**) Treated with 5 µM T-OA; (**c**) Treated with 10 µM T-OA; and (**d**) Treated with 20 µM T-OA.

Mitochondrial membrane potential (Δψ_m_) is an important parameter of mitochondrial function. It is used as an early apoptotic marker in cells. To determine whether mitochondrial damage occurs as an early event in T-OA-induced apoptosis, changes in Δψ_m_ were measured using flow cytometry with Rhodamine-123 (Rh-123), cell permeable cationic dye that preferentially enters mitochondria based on the highly negative mitochondrial membrane potential. As shown in Figure 6, the fluorescent intensity decreases from 384 to 1783, 2318 and 3078 with the increase of T-OA concentration. This indicated that T-OA was able to induce mitochondrial membrane potential depolarization in Bei-7402 cells. Depolarization of the membrane can no longer retain Rh123 and leaks out from the mitochondrial membrane to the cytoplasm, and this caused an increase in the Rh123 fluorescence intensity values in 4, 5 and 6 µM T-OA treated Bel-7402 cells as compared to the control [30,31].

### 2.7. Immumohistochemical Staining

**Figure 7:**
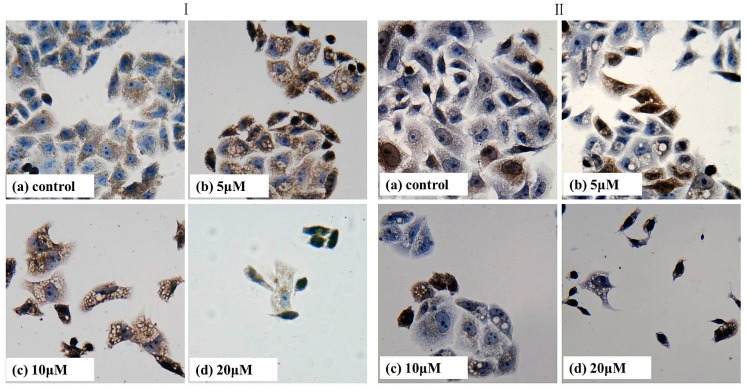
Immunohistochemical analysis of NF-κB/p65 (**I**) and COX-2 (**II**) in control and T-OA treated groups of Bel-7402 cells. (**Ia**) Control group without T-OA; (**Ib**) Treated with 5 µM T-OA; (**Ic**) Treated with 10 µM T-OA; (**Id**) Treated with 20 µM T-OA; (**IIa**) Control group without T-OA; (**IIb**) Treated with 5 µM T-OA; (**IIc**) Treated with 10 µM T-OA; and (**IId**) Treated with 20 µM T-OA. (×400).

Numerous studies have demonstrated that carcinoma cells displayed an increased expression of NF-κB/p65 and COX-2. As shown in Figure 7, p65 and COX-2 protein were expressed at a very high level in the control group. At the same time, it was obvious that the decreasing expression of p65 and COX-2 protein was accompanied with increasing concentration of T-OA. There was a visible difference between the control group and each T-OA group. Taken all together, the obtained results revealed that T-OA could prevent p65 and COX-2 activation, and exert its anticancer effect possibly through disrupting NF-κB/p65 and COX-2 signaling in Bel-7402 cells. NF-κB/p65 has proved to be associated with inhibition of cell apoptosis [32]. COX-2 could promote the metastasis of cancer cells by promoting tumor angiogenesis and inhibit the cell apoptosis [33,34]. Therefore, the result indicated that T-OA may induce Bel-7402 cells apoptosis by inhibiting the expression of NF-κB/p65 and COX-2.

**Figure 8:**
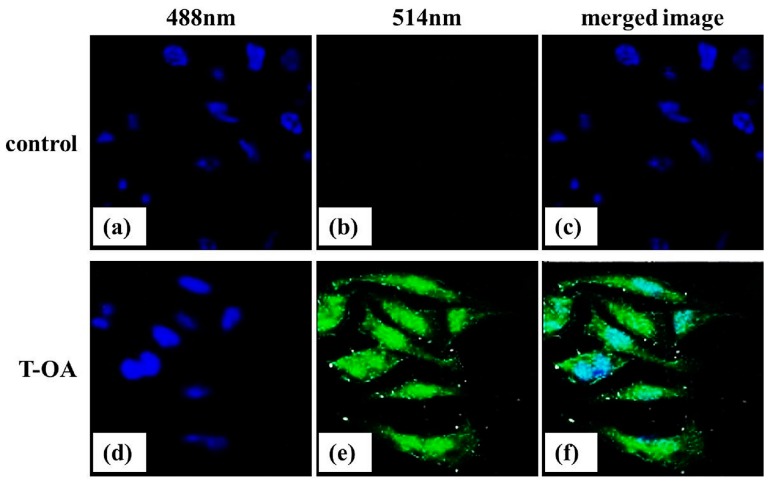
Subcellular localization of T-OA in Bel-7402 cells. (**a**) Control group at 488 nm; (**b**) Control group at 514 nm; (**c**) Merged image of control group; (**d**) T-OA fluorescent analogue treated group at 488 nm; (**e**) T-OA fluorescent analogue treated group at 514 nm; and (**f**) Merged image of T-OA fluorescent analogue treated group. (×600).

### 2.8. Subcellular Localization

Using methods of florescent tracing and confocal micrograph, we investigated the subcellular location of T-OA in Bel-7402 cells. Nuclei of Bel-7402 cells were stained with DAPI to visualize the subcellular compartments (Figure 8a,d). After being incubated with T-OA fluorescent analogue, compared with control group (Figure 8b), the green fluorescent showed that T-OA can get into Bel-7402 cells (Figure 8e). As shown in the merged image (Figure 8f), T-OA fluorescent analogue corresponded very well with the location of the nucleus, indicating that T-OA mainly accumulated at nuclei of Bel-7402 cells. When cells are stimulated with extracellular signals such as cytokines and an oxidative stressor, NF-κB translocated from the cytoplasm to the nucleus and played important roles in the anti-apoptosis effect [24]. T-OA, mainly accumulating at the nuclei of Bel-7402 cells, may relate to this phenomenon.

## 3. Experimental Section

### 3.1. General

T-OA was synthesized in our laboratory as reported previously [8]. The purity of T-OA was analyzed by Waters 2695 HPLC system (Waters Corporation, Milford, MA, USA).

The five cells lines Bel-7402, HeLa, HT-29, BGC-823 and MDCK were provided by the Chinese Academy of Medical Sciences and Peking Union Medical College (Beijing, China).

### 3.2. Cytotoxicity Evaluation

The growing cells were plated at a density of 3 × 10^4^ cells/mL and incubated in 96-well plates for 24 h (37 °C, 5% CO_2_). Then the cells were exposed to various concentrations of T-OA (0.62, 1.85, 5.55, 16.67, 50, 150 µM). After 72 h incubation, MTT solution (20 µL, 5 mg/mL) was added to each well, and the plate was incubated for a further 4 h before removing the media. Formazan crystals were dissolved with DMSO (150 µL). After mixing well, the absorbance was read at 490 nm with a BIORAD 550 spectrophotometer (BIORAD, Hercules, CA, USA). Wells containing no drugs were used as negative controls. Cell growth inhibitory rate was calculated in the following Equation (1):

(1) Inhibition% = (1 − Sample group OD/Control group OD) × 100%

### 3.3. Morphological Examination by Giemsa Staining

Giemsa staining was performed according to our previous study with minor modifications [5]. Briefly, Bel-7402 cells (3 × 10^4^ cells/mL) were seeded onto 6-well plates, and the plates were then incubated overnight (37 °C, 5% CO_2_) to allow adherence. Then cells were treated with or without T-OA (5, 10 and 20 µM) for 48 h. After washed with PBS twice, cells were fixed in ethanol for 10 min and stained with Giemsa for 5 min. Dyestuff was discarded and rinsed again three times with PBS. Morphological changes were examined using Olympus IX71 inverted microscopy (Olympus, Tokyo, Japan) with 400× actual magnification.

### 3.4. DAPI Staining

Bel-7402 cells in logarithmic growth phase were allowed to grow in 6-well plates for 24 h at 37 °C with 5% CO_2_. Then cells were treated with or without T-OA (5, 10 and 20 µM) for 48 h. After washed with PBS twice, the cells were fixed with 4% paraformaldehyde (pH 7.4) for 15 min. With an excitation wavelength of 488 nm, DAPI staining was then performed for 2 min and nuclear fragments were observed using Olympus IX71 inverted microscopy (Olympus, Tokyo, Japan) with 400× actual magnification.

### 3.5. AO/EB Staining

After the pre-processing method mentioned above. The cultured cells were stained with 50 μL of AO/EB stain (100 μg/mL) for 10 min and then the fluorescence was observed using fluorescence microscope.

### 3.6. Apoptosis Analysis

Annexin V-FITC/PI apoptosis detection kit was used according to the manufacturer’s protocol. Briefly, Bel-7402 cells (3 × 10^4^ cells/mL) were treated with or without T-OA (4, 5 and 6 µM) for 48 h. Total cells were then washed with cold PBS twice and re-suspended gently in 200 µL binding buffer. According to the manufacturer’s instructions, Annexin V-FITC and PI (YEASEN, Shanghai, China) were added into each sample. After incubated for 20 min in a dark place and then analyzed by BD FACSCanto II fluorescence activated cell sorter (BD, Franklin Lakes, NJ, USA).

### 3.7. Mitochondrial Membrane Potential Assay

Bel-7402 cells (5 × 10^5^ cells/mL/well) were seeded in 6-well culture plate and incubated for 24 h. Cells were treated with different concentration (0, 4, 5, 6 and 100 µM) of T-OA for 48 h treatment. Rh-123 (10 µg/mL) was added 100 µL before the termination of experiment, incubated at 37 °C for 30 min and thereafter washed with PBS. The pellet collected by centrifugation, was resuspended in 300 µL of PBS. The florescence intensity of Rh-123 in cells was analyzed using flow cytometer (BD, Franklin Lakes, NJ, USA).

### 3.8. Immumohistochemical Staining

Bel-7402 cells were treated with or without T-OA (5, 10 and 20 µM) for 48 h. Total Cells were fixed in 4% formaldehyde for 30 min 37 °C then washed three times in PBS. Sequently, cells were treated with 2% H_2_O_2_ in methanol for 20 min to block endogenous peroxidase activity followed by another wash and then blocked with 10% BSA for 30 min. The cells were incubated with primary antibody (anti-p65 and COX-2) at 4 °C overnight. The next day, cells were washed three times in PBS then incubated with second antibody. After being washed with PBS three times, cells were treated with SABC for 30 min at 37 °C then developed with DBA. After counterstained nuclei with haematoxylin, cells were photographed under Olympus IX71 inverted microscopy (Olympus) with 400× actual magnification.

### 3.9. Subcellular Localization of T-OA

T-OA was reacted with fluorescein isothiocyanate at 4 °C overnight. Then the bioconjugate were purified by gel filtration chromatography on a PD10 column (General Electric Company, Fairfield, CT, USA).

Bel-7402 cells grown on the cover glass were incubated with florescent labeled T-OA (10 µg/mL) at 37 °C for 1 h. DAPI (1 µg/mL) staining was then performed for 2 min as nuclei marker. Before being visualized the subcellular distribution under Olympus FV1000 confocal microscopy (Olympus), the cells were washed with PBS three times under strictly subdued light conditions. With excitation wavelength of 488 and 514 nm, subcellular Localization of T-OA was observed using oil objective lens.

## 4. Conclusions

In summary, our present study indicated that T-OA displayed greater selective cytotoxicity against the human hepatoma cell line Bel-7402 (IC_50_ = 6.36 ± 1.56 µM) than other three cancer cell lines (HeLa, HT-29, BGC-823). Moreover, at the concentration of 50 µM, the inhibition rate of T-OA against MDCK cell line was under 5%, while the inhibition rate of T-OA against Bel-7402 cells was up to 98%. T-OA showed lower renal toxicity in comparison with Cisplatin. The morphological results showed the compound caused Bel-7402 cells to present typical characteristics of apoptosis, such as nuclear condensation, nuclear fragmentation and the formation of apoptotic bodies. Furthermore, anticancer mechanism tests confirmed/revealed T-OA induced apoptosis in Bel-7402 via a pathway involving down-regulating the expression of NF-κB/p65 and COX-2. We used fluorescence microscopy to track the subcellular localization of T-OA. The result suggested that T-OA exerted its effect in the nuclei which illustrated further T-OA decreased the expression of NF-κB/p65 and COX-2. This demonstrated that T-OA has a selective apoptosis-inducing effect on the Bel7402 cells but lower renal toxicity than that of Cisplatin. Therefore, T-OA may be considered as agents with potential for development as a valuable candidate for further research.
